# Building the National Database of Health Centred on the Individual: Administrative and Epidemiological Record Linkage - Brazil, 2000-2015

**DOI:** 10.23889/ijpds.v3i1.446

**Published:** 2018-11-14

**Authors:** Augusto Afonso Guerra Junior, Ramon Gonçalves Pereira, Eli Iola Gurgel, Mariangela Cherchiglia, Leonardo Vinicius Dias, Juliano D Ávila, Núbia Santos, Afonso Reis, Francisco Assis Acurcio, Wagner Meira Junior

**Affiliations:** 1 Federal University of Minas Gerais – Faculty of Pharmacy Av. Presidente Antônio Carlos, 6627 Campus Pampulha – Cep: 31270-901 Belo Horizonte – MG – Brasil; 2 Department of Computer Science, Federal University of Minas Gerais, Belo Horizonte, Brazil; 3 Faculdade de Medicina da Universidade Federal de Minas Gerais, Av. Pres. Antônio Carlos, 6627 - Pampulha, Belo Horizonte - MG, 31270-901, Brazil; 4 Laboratory of Bioinformatics and Systems (LBS) Universidade Federal de Minas Gerais Av. Pres. Antônio Carlos, 6627 - Pampulha, Belo Horizonte - MG, 31270-901 | Brazil; 5 Ministry of Health - Esplanade of Ministries, Block G, Ground Floor; Brasília / DF - CEP: 70058-900

**Keywords:** Data linkage, record linkage, Brazilian health database, SUS deduplication

## Abstract

**Introduction:**

In Brazil, the National Health System (SUS) provides healthcare to the public. The system has multiple administrative databases; the major databases record hospital (SIH) and outpatient (SIA) procedures. Epidemiological information is collected for all populations in subsystems, such as mortality (SIM), live births (SINASC) and diseases of compulsory declaration (SINAN). Each subsystem has its own information system, which is able to provide information about consultations, clinical information and medicines dispensed. However, these systems are not linked, thereby preventing individual-centred analysis.

**Objective:**

To describe the methods and results of parameter setting that are needed to execute the probabilistic deduplication of large administrative and epidemiological databases in Brazil and to create a National Health Database Centred on the individual.

**Methods:**

This paper shows the results of a record linkage model to integrate data from SIH, SIA, SIM, and SINAN, which have different formats and attributes between them and over time. These data consist of 1.3 billion records from 2000-2015. Probabilistic and deterministic record linkages were used to deduplicate these data. The Kappa statistic and clerical review were used to ensure the quality of the linkage. The graph algorithm and depth-first search were used to generate the identifiers.

**Results:**

The deterministic deduplication process resulted in a database with 403,113,527 possible unique individuals. After the probabilistic deduplication process of the former database was performed, 159,703,805 unique individuals were identified. This result had an estimated a false positive error rate of 3.3%, and the false negative error was estimated at 12.3%.

**Conclusions:**

The National Health Database centred on the individual was generated and will allow researchers to use real-world evidence to conduct clinical, epidemiological, economic and other studies. This database represents a significant cohort, spanning 15 years of historical data and preserving patient privacy. The success of the process described will allow repeating and appending the data for future years and enable important studies to promote SUS efficiency and provide better treatments for patients.

## Introduction

### The Brazilian Unified Health System (SUS)

In Brazil, the right to healthcare is universal. The Brazilian National Health System (SUS) is funded by taxes and provides primary care, outpatient care, hospital care and medicines for a population of approximately 200 million inhabitants spread across the continental area. Citizens that do not want to wait in the queue and who can afford it purchase supplementary private health insurance.

### SUS Information Systems – Administrative and Epidemiological Control

SUS has information systems for administrative and epidemiologic control. At the individual level, the main administrative databases are the Hospital Information System (SIH) and the Outpatient Information System (SIA), which also records diagnostic procedures and information about dispensing data of medicines used in specialist outpatient services. The main epidemiological information databases are the Information System of Mortality (SIM), the Information System of Livebirths (SINASC) and the Information System on Diseases of Compulsory Declaration (SINAN).

These systems, although consistent with the administrative needs for payments and the transfer of funds or epidemiological monitoring, do not share the same personal identifier number, which generates a fragmented outlook of the health system (1).

### Challenges - the systems identify the service providers but not the patients.

One of the biggest challenges of health databases is the unambiguous identification of patients or, in other words, entity resolution. The Brazilian government developed the National Health Card (CNS) in 2001, aiming to identify each citizen. However, under Brazilian law, the SUS is obliged to provide health care to any citizen, even if he or she does not carry an identification document. As a consequence, in many situations, health unit personnel around the country create a new CNS card number for a person that might already have one.

The duplication of the CNS numbers makes it difficult to create an electronic medical record to store data accurately and follow patient trajectories and health states over time. Another important utility of the unambiguous identification of patients in big data in health is to allow performance evaluation of financed health technologies. Real-world studies conducted with big data allow decision-making to deliver better and more effective public policies.

### How to integrate different databases without a unique identifier?

Fellegi & Sunter developed a record linkage technique, which includes a deterministic and probabilistic deduplication of records (2) aiming to integrate different databases without a unique identifier (3, 4). This technique represents an important tool to link Brazilian health data, providing wider applicability than that for which they were created. Health record linkage is widely used by international and national scientific studies aiming to develop integrated databases focused on individuals (5–12). According to these authors, the probabilistic deduplication has been successful in enabling epidemiological and economic studies in the health field.

Thus, this paper aims to describe the process of building an individual-centred health database from the integration of different subsystems of SUS using record linkage techniques.

## Methods

### Study setting

This study integrated health data from SUS, including all 26 Brazilian States and the Federal District, which covers a population of approximately 200 million inhabitants. The data include records of hospital care (SIH) and outpatient care, including consultations, diagnostic procedures, and medicines dispensed for domiciliary use prescribed by specialist services (SIA), death certificates (SIM) and data from diseases and conditions of mandatory reporting (SINAN). With the exception of the SINAN (2008-2015), all the other integrated databases have records from January 2000 to December 2015.

### Description of data sources used for linkage

**Outpatient data (SIA)** include two subsystems with different attributes: the individual outpatient production report (BPAI), and the authorization of high complexity outpatient procedures (APAC). These subsystems cover all outpatient procedures, consultations, primary and secondary diagnosis codes (coded using the 10th version of the International Coding of Disease, ICD-10) and codes for performed procedures, quantity produced, and the amount paid by SUS. Also included are: medicines dispensed for domiciliary use, which are coded as procedures and personal data: patient’s full name, mother’s full name, date of birth, residential address, the city of residence, state of residence, zip code, sex, CNS number or Individual Taxpayer Registry number.

**Hospital data (SIH)**, hospitalization, covers all inpatient procedures and separations (e.g. hospital discharges, transfers, and deaths). Data include dates of admission and separation, primary and secondary diagnosis codes (coded using the ICD-10), and codes for procedures performed during the stay and amount paid by SUS. Personal data include: patient’s full name, the full name of the guardian/representative for the patient, date of birth, residential address, the city of residence, state of residence, zip code, sex, a personal document of identification or CNS number.

**Mortality data (SIM)**: includes date and cause of death and secondary contributing causes (coded using the ICD-10). Personal data include: patient’s full name, mother’s full name, father’s full name, date of birth, residential address, the city of residence, state of residence, zip code, sex and CNS number.

**Diseases and conditions of mandatory reporting and control data (SINAN)**: mandatory notifications for physicians to notify any mandatory disease or condition under surveillance. Data include disease, date of onset, diagnosis method and organism serotype (where appropriate). Also included are data on routine microbiology tests performed and personal data: patient’s full name, mother’s full name, date of birth, residential address, the city of residence, state of residence, zip code, sex and CNS number.

### Study Design

We considered a viable patient name key for the quality of the probabilistic de-duplication. Databases originated from mortality notification (SIM) and hospitals (SIH) are more prone to receive records from emergency situations, such as from patients without identification who have been involved in an accident, homeless people without documents or new-borns that do not yet have names. We found 12.0% and 8.2% of records without suitable identifiers in SIH and SIM, respectively. Since SIA and SINAN collect data mostly from scheduled visits, the percentage of non-viable records was 2.5% in each.

To preserve the confidentiality of clinical events, medical treatments performed and causes of death, we separated the databases into distinct tables. In one table, we gathered personal identification data, containing patient’s full name, mother’s full name, father’s full name, date of birth, sex, CNS number, taxpayer registry number, residential address, the city of residence, state of residence and zip code from all databases. Since all databases do not present all these attributes and even when they do, they might be unfilled, we declared missing attributes as null. The health events of each database were stored in separated tables without personal data but linked by a unique code. The researchers only had access to the table with personal data.

**Figure 1: Model proposed to create the Brazilian National Database of Health centred on the individual:**
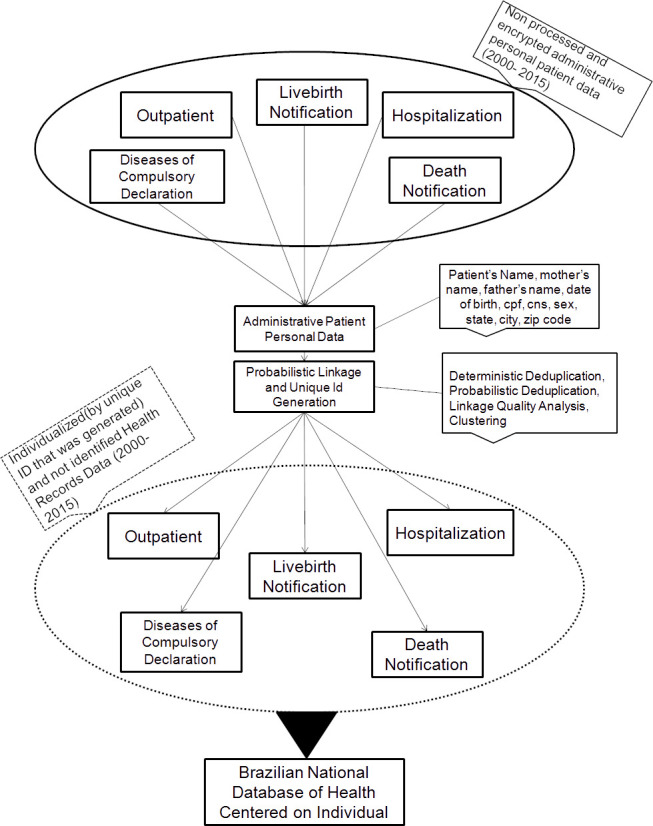


We designed a process, shown in Figure 1, in which we proposed to integrate these different data sources (administrative and epidemiological), and prepared it to execute the linkage to the creation of Brazilian National Database of Health Centred on Individual.

The Brazilian National Database of Health centred on the individual was constructed following the steps: 1) Pre-processing; 2) Deterministic deduplication intra-system; 3) Deterministic deduplication inter-system; 4) Probabilistic Deduplication; 5) Linkage quality analysis and Classification; and 6) Clustering.

### Pre-processing

Cleaning and standardization are part of the data quality step. This approach is used to increase the effectiveness of deduplication through the exclusion of invalid names and standardization of fields to have the same semantics and to add value to the probabilities.

In the cleaning and standardization stage for each attribute in the table of personal data, we carried out a standardization and specific cleaning. For the sex attribute, we standardized values “M” for male individuals and “F” for female individuals. For the name attributes (patient’s name, mother’s name and father’s name), we removed abbreviations, special characters, accented characters, line breaks, spacing, connectives, and preposition. In addition, we generated an auxiliary table with 21,818 invalid names found in records such as “NEWBORN, STILLBIRTH, NB, INVALID NAME and UNKNOWN NAME”.

For the CPF number attribute, we removed invalid values with non-numeric characters or less than the standard 11 digits, and we have done a cross-checking with the national Brazilian taxpayer database to certify if the CPF number exists.

For the CNS number attribute, we removed invalid values with non-numeric characters or less than the standard 15 digits and performed a cross-checking with the national database of health cards (CADSUS) to certify if the CNS number exists. We performed verification, by the similarity of the names, with a minimum threshold of 90%, to recovery the definitive CNS number for patients who had more than one number. As the same patient can present several different CNS numbers, due to the preliminary registration in the health units around the country, the final number of the CNS is only assigned univocally after the DATASUS services from Ministry of Health perform binding procedure per patient.

For the date of the birth attribute, we removed invalid dates and dates of birth prior to 1 January 1881. For the attributes of geolocation, we cross-checked with the national database of cities and states from the Brazilian Institute of Geography and Statistics. Zip codes were cross-checked with the national database of the Brazilian Post and Telegraph Company. In both situations, if we did not find these values, we declared them as NULL.

### Deterministic Deduplication: Intra-system and Intersystem

With the use of the statistical and mathematical framework for record linkage (2), we carried out the deterministic deduplication of records intra-system when creating the table with personal identification data for each data source to preserve the characteristics of the original system and to increase the quality of the linkage. After this step, we performed an inter-system deterministic deduplication of records generating the Intra- and Inter-system deterministic table with all personal identification data.

### Probabilistic Deduplication

Thus, with the intra-system and inter-system deterministic table, we started the probabilistic deduplication stage. At this stage, the steps were parameter estimation, the creation of blocking strategies and comparison of records.

We established parameters through a sample estimation of records (13); the other alternative would be the Expectation Maximization algorithm (2, 14). In both methods, the values are distributed in two main variables: *m* and *u*. The value *m* is the value measured for an attribute comparison when the pair is considered true and the attribute agrees on that pair. The value *u* is the value measured for an attribute comparison when the pair is considered false yet the attribute still does agree on this pair. The probability of *u* is similar to the false-positive proportion (1-specificity). The probability of m is similar to the sensitivity, i.e., the proportion of real positives that were correctly identified.

The weight given to an attribute of a pair is $wi=\log_{2}\frac{m}{u}$ if the attribute agrees and $wi=\log_{2}\frac{1-m}{1-u}$ if the attribute disagrees. The score of a pair is given by the sum of all weights *wi* of this pair. The logarithm of base two is used, so that the concordance/discordance weights can be added, generating a score for each compared pair. In addition, it is necessary to establish weights when in the same attribute in one of the records of the pair compared its value is null. This weight is defined by $\frac{m+u}{2}$ for missing data (8).

For the name attributes as well the attributes of geolocation we used the calculation of the concordance weight based on frequency tables, because their values have an unequal frequency distribution. The calculation of the weights based on frequency tables was extracted from the database with $\log_{2}\frac{1}{\text{relative frequency}}$ as the assigned weight. Only those records with a relative frequency greater than 0.00001% were kept in the frequency table, and if the attribute value was not found in the frequency table, it had its weight assigned to the highest possible weight, i.e., when the relative frequency was less than 0.00001%.

Blocking is the step responsible for making computationally possible the execution of probabilistic deduplication of records. In an ideal world, it would be important to compare all the records in the database with each other, but this is still not possible, given the limitations of memory and processing. The blocking is created to avoid unnecessary comparisons; for example, Mary, female, does not have to be compared to William, male. Thus, the blocking divides the “Marys” and the “Williams” into separate groups of records. To allow the creation of “approximate blocks” for strings the Soundex algorithm (15) can be used. Soundex transforms names through phonetics; for example, names such as “Robert” and “Rupert” are considered the same because they have the same Soundex number and can be misspelled. This approach increases the number of records in the blocks and allows grouping records that are susceptible to typing errors.

The comparison stage is made for each attribute for all pairs in each block. For names attributes, we used Jaro Winkler Distance (13) algorithm of string similarity comparisons with a threshold of 90%. The comparison result was a list of pairs and the respective score. The scores were classified into two cut-off points: the zone below, in which pairs below a threshold were considered false, and another above, in which pairs above a threshold were considered true. Pairs with a score between the two cut-offs were considered dubious pairs. These “zones” were previously defined based on the parameters.

### Linkage Quality Analysis

To evaluate the reliability of zones established by the previous classification where pairs are considered true, false or dubious, we performed a linkage quality analysis. At this step, we performed a clerical review to inspect a random sample of pairs in each zone. This approach allowed us to verify the effectiveness of the cut-off score, which was previously chosen, in the deduplication and the need for a revision of parameters, blocking strategies or new scores for each zone.

### Clustering

Clustering is the stage responsible for turning pairs into a set of records that belong to the same person. For example, given a pair of records formed by (A, B) once the pair is considered true, i.e., A = B, the records A and B will have the same unique identifier. If the pair (B, C) is considered true, the records A, B and C, by transitivity, will have the same identifier record.

### Software and Hardware

There are many tools/software available for record linkage (16–19). We tested some of these alternatives, but due to the large volume of data, we had trouble with computer performance. Therefore, we decided to adapt/implement codes as described for each stage. For the stages: “Pre-Processing, Deterministic deduplication (intra-system and inter-system) and Linkage quality analysis” we developed MySQL scripts, including a web-based tool for clerical review. To perform the probabilistic deduplication we used Pareia(16) code in C language, as it is Brazilian free software developed in one of our former projects. Pareia showed a capacity of parallel and distributed processing for big data. We also implemented the DFS algorithm in Java for Clustering stage.

We executed all tasks of this linkage in a server running SUSE-Linux 12.0 with processor architecture x86_64, 2 sockets, model Intel(R) Xeon(R) CPU E5-2690 v2 @ 3.00 GHz, 10 cores per socket, 2 threads per core, 25600K cache L3, which means a effective power of 40 processors cores, Ram memory 384GB, DDR3, Synchronous LRDIMM, and a 1866-MHz hard drive with a 4-TB type SSD.

### Ethical approval

The Research and Ethics Committee of the Universidade Federal de Minas Gerais approved this research (44121315.2.0000.5149).

## Results

We performed cleaning and standardization to ensure data quality for the deduplication process. At this step, we excluded invalid names, dates, CPFs, and CNSs, and we standardized the attributes according to the methods.

After processing the deterministic deduplication intra-system for each database, we gathered all data into the table of personal identification for the period of 2000-2015 leading to 1,085,851,567 records, as shown in Table 1. We used these records to process the deterministic deduplication inter-system and obtained 403,113,527 records.

**Table 1: Databases received from the Ministry of Health in the period of 2000-2015:** (*)2008-2015

Databases	Number of records

Hospitalization Information System (SIH)	188,512,557
Outcomes Information System (SIA (APAC;BPAI)	869,709,353
Mortality Information System (SIM)	16,635,462
Diseases and conditions of mandatory reporting and control data (SINAN)*	10,994,195

TOTAL	1,085,851,567

The probabilistic deduplication process begins with an estimation of the probabilities using a sampling strategy. We randomly sampled 1,000,000 records and carried out a probabilistic deduplication with parameters used in the paper (22). At this step, we recalculated the values of “*m*”, “*u*” and “missing”. We executed this process repeatedly and by the third round, it was possible to see a trend towards stabilisation of the probabilities. The final values found are shown in Table 2.

**Table 2: Values of m and u, and respective weights of agreement and disagreement, for the probabilistic deduplication of records.** (*)The amplitude of variation of agreements’ weight for these attributes was calculated through frequency tables.

Attribute	M	U	Agreement Weight	Disagreement Weight	Missing Weight

Patient’s name	0.8523	0.0046	7.5377 - 14.2942*	-2.7528	2.3925
Mother’s name	0.8448	0.8222	0.0391 - 14.2880*	-0.1959	-0.0784
Father’s name	1.0000	1.0000	0.0000 - 14.2907*	0.0000	0.0000
Sex	0.9868	0.9312	0.0836	-2.3779	-1.1472
CPF	0.9999	0.9102	0.1356	-0.1215	0.0000
CNS	0.9389	0.9414	-0.0038	0.0590	0.0276
Date of Birth	0.2132	0.1842	0.2109 - 14.2880*	-0.0522	0.0794
State	0.9872	0.9694	0.0263 - 10.3754*	-1.2610	-0.6173
City	0.9760	0.8896	0.1338 - 14.2886*	-2.2041	-1.0352
Zip code	0.1979	0.0008	-2.3428 - 14.2880*	-0.3170	3.8546

To process the blocking step, we used the standard blocking algorithm (2), which has been set up by the data analysis with previous studies published by researchers (20) and our empirical experience working with SUS data. We established nine blocking strategies, as shown in Table 3, with the respective number of pairs generated. For the attributes of patient’s name, mother’s name and father’s name, we also used the Soundex algorithm with an adaptation for Brazilian names (3).

**Table 3: Blocking strategy numbers of pairs generated:** 

ID	Blocking Strategy	Pairs

1	Cpf	12,480,286
2	Cns	151,730,408
4	patient’s first name, patient’s middle name, patient’s last name, date	486,325,426
4	mother’s first name, mother’s last name, date	1,215,062,093
5	patient’s name, patient’s last name, mother’s last name, month, year, sex	1,722,402,253
6	patient’s name, patient’s last name, mother’s first name, mother’s last name, zip code	1,506,043,390
7	patient’s name, patient’s last name, state, day, month, sex	2,535,059,431
8	patient’s name, patient’s last name, state, day, year, sex	2,830,748,051
9	patient’s name, patient’s last name, state, month, year, sex	6,304,252,194

The execution time of the blocking steps and comparison were 23 hours and 47 minutes, respectively. The comparison step involved approximately seven billion pairs, once the algorithm was used, to avoid comparing the same pairs generated by various blocking strategies. Additionally, due to restrictions on disk space, only pairs with a score higher than -5 were stored on the disk, which made it difficult to calculate the real number of pairs compared.

**Figure 2: Score distribution:**
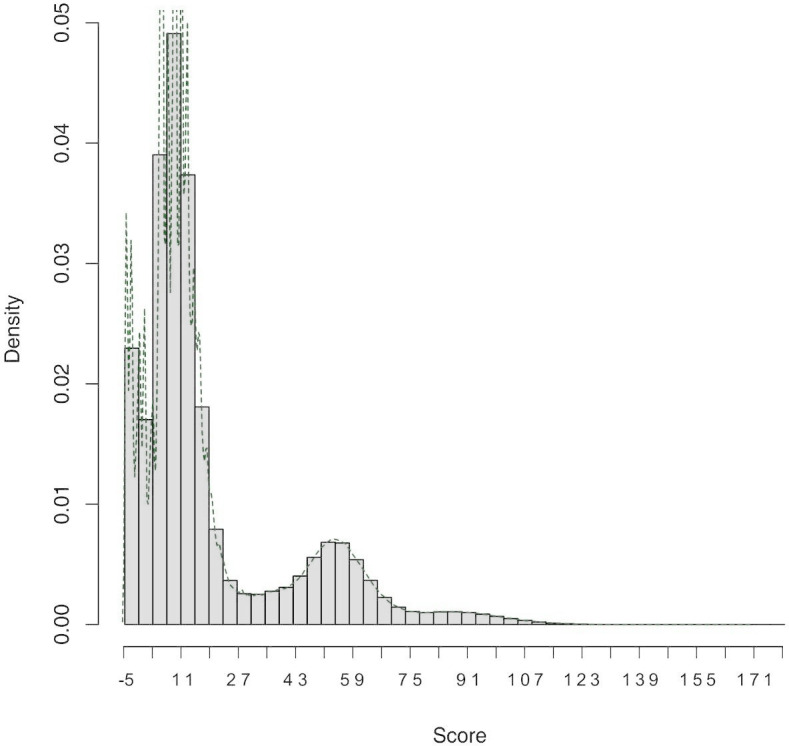


The distribution of the scores of the pairs compared is shown in Figure 2. We considered pairs with scores between 20 and 34 to be the “grey zone”. Pairs with scores lower than 20 were considered false, and pairs with scores higher than 34 were considered true.

In the linkage quality analysis, we separated the amplitudes of the scores into 5-point ranges, resulting in 28 random samples with 432 pairs each, totalling 12,096. Thus, the first range has scores between 135-140, and the last range between -5 and 0. In a clerical review, those pairs were manually inspected and classified as correct or incorrect by two independent reviewers.

The Kappa statistic was used to assess agreement between the two reviewers (20). This statistic was not used to evaluate the agreement of the reviewers with the deduplication since the purpose of this review was not to compare the method of probabilistic deduplication of records with the manual procedure but to obtain an estimate of the quality inherent to the deduplication. The clerical review showed a mean agreement between the ranges of 97.4% of cases (Kappa = 0.952).

**Figure 3: True pairs x full ranges and Figure 4: True pair x expanded ranges:**
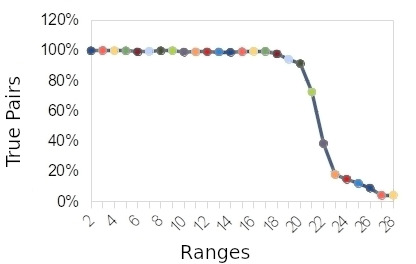


**Figure 4: True pair x expanded ranges:**
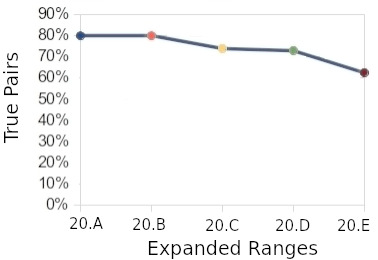


Considering the reviewers’ decision, the frequency of true pairs for each range evaluated is shown in Figure 3. At range 20, scores between 30 and 35, it was possible to see the decrease for true pairs. To determine the exact score, the range 20 was expanded into another 5 ranges (20.a, 20.b, 20.c, 20.d, and 20.e), Figure 4. These new ranges were split by only one score point. The first range (20.a) had scores between 34-35, and the last (20.e) had scores between 29 and 30. More than 2,160 random pairs were totalled in order to obtain a final decision on the cut-off score.

In the range “20.d”, we can see the decrease starting for true pairs. This range was defined as a cut-off point, and a score of 30, on the lower side, was selected. By taking this score, we calculated the estimated error. For the false positive error, the estimated value was 3.3%, while the false negative error was estimated at 12.3%. The false-negative error was greater because the number of pairs compared was smaller than the number of pairs that should be compared (all versus all). This limitation occurred due to computational constraints.

We performed the clustering step with an adaption of the depth search graph algorithm (21). This algorithm generated the unique identifiers sequentially, and MySQL scripts were used for insertion into databases. After clustering, it was possible to identify some anomalous clusters with a large number of records. These clusters were extracted and resubmitted to the deduplication process with new probabilities adjusted.

Thus, the national database on individual-centred health was generated with 159,703,805 patients who were considered unique.

## Discussion

The Brazilian National Health System (SUS) provides free access to administrative and epidemiological information databases (Tabnet), and its web-based tools are widely used for research and as health indicators. Although a few studies have integrated these systems, the use of these data generates a fragmented outlook. The probabilistic deduplication of records performed in this work allows the generation of epidemiological indicators that are linked to morbidity, economic burden, and mortality. In addition to other possibilities, the integration enables outcome research and the pharmacoeconomic and performance assessment of health technologies, delivering better and more effective public policies.

Data cleaning and standardization have proven to be relevant and laborious steps in the process, given the high frequency of inconsistent, incomplete, or misspelled data (8). In fact, administrative databases were not designed specifically for research purposes. Thus, it was necessary to create a model that would allow the inclusion of administrative data from different sources.

Deterministic deduplication was important to reduce unnecessary computation efforts on the probabilistic step, as this process decreases the number of records involved. This reduction is explained by the fact that the databases in Brazil are not integrated internally or externally. Additionally, this approach preserves the semantics of the original subsystem, from which the data originate, which may improve linkage quality.

In probabilistic deduplication, the probability values (m, u and missing) determine where to set the match/non-match thresholds. These probabilities balance the result, aiming at an acceptable positive predictive value (precision). Sampling methods show good results; however, they are costly because we must run them repeatedly until we achieve weights that maximize precision.

Table 2 shows the estimated values for the probabilities of the probabilistic deduplication of records. Some probabilities were the opposite of what we expected; for example, the predictive value of CNS as a unique identifier was low. This value occurred because of the SUS policy of creating provisional numbers when patients do not present one. With the Tax Payer Number (CPF), this problem does not occur, as the federal authority responsible for this database only provides a definitive number for each citizen. However, CPF frequency in SUS databases is low.

To be able to process the large-scale data involved, we carried out several blocking strategies that ended up influencing the result. By definition, blocks require an equality in the pairs between the attributes of the block. As we used birth date frequently in the blocking strategy, the birth date was reflected in the u value. Sex is another attribute of a high u value and also has a relationship with the blocking strategy. Proper Portuguese names used in Brazil that end with the letter “A” are usually for females, and those that end with “O” are usually for males. We used the attribute “patient’s name” in six of the nine blocking strategies, and in these comparisons, sex matched, even though the pair was false.

The estimation of the errors gives a general idea of the possible problems, and the need to evaluate the records before carrying out epidemiological and economic studies. Inconsistent clusters generated by false positive and false negative errors must be considered and excluded in future research. In addition, given the tight blocking criteria used, it is quite possible that there was a greater false-negative pair rate. It was a trade-off, to make the record linkage possible; we know that we left some false-negative pairs behind, but we tried to use the best attributes as possible to increase the linkage quality.

In the clustering step, the anomalous clusters were the ones with common names in Brazil and/or records related to large cities. In these cases, we decided to deduplicate and detach them to ensure better matching results, as they demanded specific probabilities.

To achieve the optimal execution time described, with a heuristic approach, we executed the process many times. In each execution, we improved the scripts and codes to increase the software performance considering our hardware limitations.

## Limitations

Despite the good results of record linkage, it is not guaranteed that the integrated data are suitable for all possible research scenarios. The real limitations of this linkage for epidemiological and economic studies can only be effectively evaluated by building longitudinal complex cohorts and comparing results with other references. One example is when analysing kidney survival transplantation; it is well established that for several reasons, deceased donors have worse results than live organ donors. In a scenario of finding the opposite results in a hypothetical cohort from this database, researchers should be careful and look for other sources of information to confirm results. Once this kind of external validation is carried out, we will know the usefulness of the developed integrated database.

Another important limitation comes from the way the data were inputted in the databases by several different services across the country and on the semantics of each available attribute. Researchers need to understand the data provenance when seeking to use the data for the specific research purposes.

## Conclusion

Our deduplication process was successfully completed and generated a database focused on the individual, which will allow researchers to perform clinical, epidemiological and economic studies, in addition to allowing better decision-making for the Brazilian Health System (SUS). The software and hardware were exhaustively tested, aiming towards the integration of the databases. This process allowed us to understand the difficulties of executing the probabilistic deduplication process of Big Data. This integrated database represents a significant advance comprising 16 years of historical health data while preserving patient privacy. The challenges for future work are the continuous integration of new records with the development of incremental deduplication on this patient-centred database.

## Abbreviations

CadSUS	Single Health Record System
CPF	Individual Taxpayer Registry number
CNS	National Health Card Number
BPAI	Individual Outpatient Production Periodicals
SIA	Outpatient Information System
SIH	Hospital Information System
SIM	Information System of Mortality
SINAN	System on Diseases of Compulsory Declaration
SINASC	System of Livebirths
